# Partial Truncation of the C-Terminal Domain of PTCH1 in Cancer Enhances Autophagy and Metabolic Adaptability

**DOI:** 10.3390/cancers15020369

**Published:** 2023-01-06

**Authors:** Begoña Caballero-Ruiz, Danai S. Gkotsi, Hattie Ollerton, Cintli C. Morales-Alcala, Rosa Bordone, Georgia M. L. Jenkins, Laura Di Magno, Gianluca Canettieri, Natalia A. Riobo-Del Galdo

**Affiliations:** 1 Department of Molecular Medicine, Sapienza University of Rome, 00161 Rome, Italy; 2 School of Molecular and Cellular Biology, Faculty of Biological Sciences, University of Leeds, Leeds LS29JT, UK; 3 Leeds Institute for Medical Research, School of Medicine, University of Leeds, Leeds LS29JT, UK; 4 Institute Pasteur Italy-Cenci Bolognetti Foundation, 00161 Rome, Italy; 5 Leeds Cancer Research Centre, University of Leeds, Leeds LS29JT, UK; 6 Astbury Centre for Structural and Molecular Biology, University of Leeds, Leeds LS29JT, UK

**Keywords:** Hedgehog, PTCH1, ATG101, autophagy, glycolysis, cancer

## Abstract

**Simple Summary:**

We have recently reported that the cytosolic C-terminal domain (CTD) of the tumour suppressor PTCH1, the Hedgehog proteins receptor, interacts with the autophagy-related protein ATG101 and impairs autophagic flux. In this study, we identified the interaction region and found that it is absent in a subset of colorectal, stomach and endometrial cancers. We demonstrated that truncation mutants lack the ability to constraint autophagy, resulting in a proliferative advantage and reduced sensitivity to autophagy inducers or glycolysis inhibitors in cell lines harbouring endogenous PTCH1 CTD mutations compared to isogenic cells expressing wild-type PTCH1. In summary, this study highlights the importance of the PTCH1-ATG101 interaction in the regulation of basal and stimulated autophagy and the metabolic flexibility in cancer cells.

**Abstract:**

The Hedgehog receptor, Patched1 (PTCH1), is a well-known tumour suppressor. While the tumour suppressor’s activity is mostly ascribed to its function as a repressor of the canonical Smoothened/Gli pathway, its C-terminal domain (CTD) was reported to have additional non-canonical functions. One of them is the reduction of autophagic flux through direct interaction with the Unc-51, like the autophagy activating kinase (ULK) complex subunit autophagy-related protein-101 (ATG101). With the aim of investigating whether this function of PTCH1 is important in cancer cell fitness, we first identified frameshift mutations in the CTD of PTCH1 in cancer databases. We demonstrated that those mutations disrupt PTCH1 interaction with ATG101 and increase autophagic flux. Using deletion mutants of the PTCH1 CTD in co-immunoprecipitation studies, we established that the 1309–1447 region is necessary and sufficient for interaction with ATG101. We next showed that the three most common PTCH1 CTD mutations in endometrial, stomach and colon adenocarcinomas that cause frameshifts at S1203, R1308 and Y1316 lack the ability to interact with ATG101 and limit autophagic flux, determined by bafilomycin A1-sensitive accumulation of the autophagy markers LC3BII and p62. We next engineered PTCH1 indel mutations at S1223 by CRISPR/Cas9 in SW620 colon cancer cells. Comparison of two independent clones harbouring PTCH1 S1223fs mutations to their isogenic parental cell lines expressing wild-type PTCH1 showed a significant increase in basal and rapamycin-stimulated autophagic flux, as predicted by loss of ATG101 interaction. Furthermore, the PTCH1 CTD mutant cells displayed increased proliferation in the presence of rapamycin and reduced sensitivity to glycolysis inhibitors. Our findings suggest that loss of the PTCH1-ATG101 interaction by mutations in the CTD of PTCH1 in cancer might confer a selective advantage by stimulating autophagy and facilitating adaptation to nutrient deprivation conditions.

## 1. Introduction

PTCH1 is a 12-transmembrane protein that serves as the receptor of Hedgehog (Hh) glycoproteins, secreted ligands that activate the Hh signalling pathway [1]. In the absence of Hh ligands, PTCH1 exhibits a constitutive activity that represses the G protein-coupled receptor Smoothened (SMO) and prevents activation of the Glioma-associated oncogene (GLI) family of transcription factors, which have been implicated in tumourigenesis and cancer progression in many malignancies [1,2]. The binding of Hh proteins to PTCH1 block its inhibitory activity, resulting in the de-repression of SMO and activation of GLI-dependent transcription. Thus, loss of PTCH1 results in constitutive activation of the canonical Hh pathway, as illustrated by loss-of-function mutations found in over 80% of basal cell carcinomas of the skin and in the majority of Shh-type medulloblastomas [3]. PTCH1 is, therefore, a tumour suppressor gene.

In addition, PTCH1 has SMO/GLI independent functions that are collectively called “non-canonical type I” Hh signalling [4]. Among them is PTCH1′s ability to block autophagy completion [5]. Autophagy is a homeostatic catabolic process that occurs as a response to an array of biological functions that include, amongst others, adaptation to nutrient-depleted conditions, oxidative stress and cellular remodelling during development and differentiation [6]. Dysregulation of autophagy has been linked to different human diseases, including degenerative disorders, inflammatory conditions and cancer. PTCH1 blocks autophagy through the interaction of its cytosolic C-terminal domain (CTD) with ATG101 [5]. ATG101 is an essential subunit of the ULK complex in mammals, composed of ULK1, FAK family interacting protein of 200 kDa (FIP200), ATG101 and Autophagy-related protein 13 (ATG13). The ULK complex integrates signals activated by nutrient starvation or growth factor signalling to stimulate autophagy [7]. While PTCH1 interacts with the ULK complex through ATG101, it does not affect the initial steps of autophagy but reduces the formation of autolysosomes, causing the accumulation of autophagic markers and undigested cargo. We showed that the inhibition of autophagic flux triggered by PTCH1 is unaffected by SMO inhibitors or SMO deficiency despite being regulated by Sonic Hedgehog (SHH), demonstrating that it is a type I non-canonical Hh signalling output [5]. Our studies revealed that both the CTD of PTCH1 and ATG101 were strictly necessary for the inhibition of autophagic flux, which highlights the biological importance of this interaction. The CTD also regulates PTCH1 ciliary localisation and degradation. The most proximal 35–40 residues of the CTD are important for the ciliary localisation of PTCH1, necessary for its repressor activity over the canonical Hh pathway [8]. However, most of the CTD, including Y1316 and K1426, is dispensable for canonical Hh signalling but regulates both PTCH1′s basal turnover and SHH-stimulated degradation [8,9]. The most distal region of the CTD is also important for the recruitment of a pro-apoptotic complex containing DRAL/TUCAN1/pro-caspase-9 that induces cell death in the absence of SHH [9,10].

Following our findings that PTCH1 reduces autophagic flux through its CTD, we hypothesised that its interaction with ATG101 is important for the tumour suppressor activity of PTCH1, which is mainly ascribed to its function to reduce GLI1 levels via regulation of the canonical Hh pathway. Since autophagy provides biosynthetic building blocks to sustain cancer growth and exerts a protective role by degradation of damaged organelles [11,12], the loss of an autophagy-limiting function should increase cancer cell fitness. If this is the case, mutations in the CTD that jeopardize the binding of ATG101 should impair the tumour suppressor function of PTCH1.

Here, we report that the interaction of PTCH1 with ATG101 occurs through the region encoding amino acids 1316–1447. This region is absent in 5–13% of human cancers, specifically in colon, stomach and endometrial carcinomas, due to indel mutations in exons 22–23 encoding the CTD. These mutations lead to loss of interaction with ATG101 and increase basal and stimulated autophagy. Proof of principle was obtained by engineering a truncation in PTCH1 in two colon cancer cells by CRISPR/Cas9. Both mutant cell lines exhibited upregulated autophagic flux, reduced apoptosis and increased proliferation in the presence of autophagy inducers compared to isogenic cells expressing wild-type PTCH1. This study suggests that partial truncation of the PTCH1 CTD in epithelial cancers results in partial loss of PTCH1′s tumour suppressor activity through dysregulation of autophagy.

## 2. Materials and Methods

### 2.1. Reagents

LentiCRISPR v2 vector was a gift from Feng Zhang (Addgene plasmid 52961; http://n2t.net/addgene:52961, accessed on 10 June 2017; RRID:Addgene_52961). Bafilomycin A1 (cat. 1334) was from Tocris Bioscience (Bristol, UK). Acrylamide: bisacrylamide (19:1) solution (cat. 1610154) and TEMED (cat. 1610801) were from BioRad (Watford, UK). 2-deoxyglucose (cat. D8375), 3-bromopyruvic acid (car. 16490) and rapamycin (cat. R0395) were from Merk Life Science (Gillingham, UK). EBSS (cat. 24010-043) was purchased from Life Technologies (Carlsbad, CA). All other reagents were research-grade and purchased from Fisher Scientific (Loughborough, UK) or Merck Life Science (Gillingham, UK).

### 2.2. Genomic Dataset

Publicly available datasets were analysed in this study. We searched for cancer types with the highest proportion of PTCH1 mutations through the Genomic Data Commons (GDC) portal of the National Cancer Institute (https://portal.gdc.cancer.gov, accessed on 30 May 2022). Identification of mutations in the PTCH1 CTD (comprising residues 1176–1447) was performed in the top three individual project datasets with the highest overall number of PTCH1 mutations: uterine corpus endometrial cancer (TCGA-UCEC), stomach adenocarcinoma (TCGA-STAD) and colorectal cancer (TCGA-COAD).

### 2.3. Cell Lines

HEK293 cells (cat. CRL-1573) and SW620 (cat. CCL-227) were purchased from the American Type Culture Collection (Manassas, Virginia) and tested periodically for mycoplasma contamination.

### 2.4. Cell Culture

HEK 293 and SW620 cells, both parental and CRISPR/Cas9 engineered, were maintained in DMEM (Merck, cat. D6429) containing 10% fetal bovine serum (Gibco cat. 10,270,106 or Merck cat. F7524) and penicillin/streptomycin in a humidified 5% CO_2_ incubator. In preparation for transfection, HEK 293 cells were seeded in antibiotic-free media into culture dishes of different areas and transfected with Lipofectamine 2000 (Life Technologies, Carlsbad, CA, USA; cat. 11668030) when they reached 80–90% confluency, following the manufacturer’s instructions.

### 2.5. Genome Editing and Selection

HEK 293 cells were engineering by CRISPR/Cas9 to introduce a truncation in the *PTCH1* coding sequence after S1223 using a guide RNA (gRNA) targeting the region of interest (5′-CTGGGAACTATACTCCGAGT) or an irrelevant scrambled sequence (5′-GCACTACCAGAGCTAACTCA). gRNA was designed using the Benchling online tool (https://www.benchling.com/, accessed on 5 November 2018). Complementary oligonucleotides were annealed and inserted into pLentiCRISPR-v2 (Addgene cat. 52961). Cells were transfected with the PTCH1-targetting or scrambled construct, followed by puromycin selection for 72 h. Puromycin-resistant clones were obtained from single cells by the limiting dilution method. Individual colonies were sequence-verified and expanded in a complete growth medium. 

### 2.6. Generation of PTCH1 Truncation Mutants

Myc-tagged PTCH1 S1203*, R1308* and Y1316* were generated by site-directed mutagenesis of wild-type myc-PTCH1 in pCS2-MT using QuikChange II XL Site-Directed Mutagenesis Kit (Agilent), following the manufacturer’s instructions. The following HPLC-purified primers were used: PTCH1 S1203stop (forward: 5′- CTGAGCCACCCCCCTGAGTGGTCCGCTTCGC; reverse: 5′- GCGAAGCGGACCACTC-AGGGGGGTGGCTCAG), PTCH1 R1308stop (forward: 5′- CAGGGACCCCCCCTGA-GAAGGCTTGTGG; reverse: 5′- CCACAAGCCTTCTCAGGGGGGGTCCCTG) and PTCH1 Y1316stop (forward: 5′- CCACCCCTCTACTGACCGCGCAGAG; reverse: 5′- CTCTG-CGCGGTCAGTAGAGGGGTGG).

### 2.7. Quantitative PCR

Total RNA was isolated with RNeasy Mini Kit (QIAGEN, Manchester, UK; cat 74104) and quantified by A260 nm/A280 nm in a Nanodrop. Synthesis of cDNA was performed using 1 μg RNA with iScript cDNA Synthesis Kit (BioRad) using hexarandom primers following the manufacturer’s instructions. Real-time quantitative PCR was performed using 1–5 μL of cDNA and target-specific primers (PTCH1 forward: 5′- *CGATGGAGTCCTTGCCTACAA*; reverse: 5′- *CCACCAGACGCTGTTTAGTCA;* RPL32 forward: 5′- *CCCTTGTGAAGCCCAAGATC*; reverse: 5′- *TCTGGGTTTCCGCCAGTTAC*) with the SsoFast EvaGreen Supermix (BioRad) with a CFX Connect Real-Time PCR Detection System (BioRad) thermocycler. Amplification was quantified and expressed as a fold using the ΔΔCt method.

### 2.8. Co-Immunoprecipitation

HEK293 were plated in a 10 cm culture dish and transiently transfected using Lipofectamine 2000 using 20 μL transfection reagent and 8 μg total DNA. Since the PTCH1 truncation variants are more stable, to achieve comparable expression levels, only 4 μg PTCH1 variants plus 4 μg pCS2 empty vector were transfected, compared to 8 WT PTCH1. For co-immunoprecipitation (co-IP) assays, two different plasmids were used in the same reaction (4 µg of each plasmid). Twenty-four hours after transfection, cells were washed with 2 mL of ice-cold 1× PBS and scraped in 700 µL of co-IP lysis buffer (50 mM Tris HCl pH 7.5, 150 mM NaCl, 1% NP-40, 0.05% Sodium deoxycholate, 1 mM EDTA, 2.5 Mm MgCl_2_) supplemented immediately before use with 1× Proteoloc protease inhibitor cocktail (Gentaur, London, UK; cat. 44204), 0.2 mM PMSF and 1 mM DTT. Lysates were incubated at 4 °C for 30 min with rotation and then centrifuged at 16,000× *g* for 15 min at 4 °C to pellet cell debris. The supernatant was transferred to another tube and 200 µL set apart as whole cell lysate (WCL) and mixed immediately with 40 µL of 6x Laemmli buffer. The rest of the supernatant was incubated with 2 μg primary antibody, either HA-tag (Proteintech, Manchester, UK; cat. 66006-2-Ig) or myc-tag (Proteintech; cat. 16286-1-AP), at 4 °C with rotation for 1.5 h, followed by the addition of 30 µL Dynabeads Protein G (Thermo Fisher; cat. 10003D) for an additional 1 h incubation at 4 °C with rotation. A magnetic rack was used to collect and wash the beads three times with 1 mL of co-IP lysis buffer. Immunoprecipitates were extracted from the bead by the addition of 18 µL of 2× Laemmli buffer. Both beads and WCL were incubated on heat block at 45 °C for 25 min and stored at −80 °C for a maximum of 1 week for Western blot analysis.

### 2.9. Western Blotting

Cells were washed once in ice-cold PBS at the end of the treatments and scrapped directly in 1× Laemmli buffer (Sigma-Aldrich S3401). For ACC and AMPK phosphorylation, cells were lysed in SDS-urea (50 mM Tris-HCl, 2% SDS, 10% glycerol, 10 mM Na4P2O7, 100 mM NaF, 6 M urea, 10 mM EDTA) and protein concentration was quantified in a Nanodrop, followed by the addition of 2× Laemmli buffer. Cell lysates were sonicated and heat denatured at 45 °C for 30 min to prevent aggregation of transmembrane proteins. Cell lysates, or immunoprecipitates, were separated together with a protein marker (Precision Plus Protein Dual Color Standards (BioRad; cat. 1610374) by SDS-polyacrylamide gel electrophoresis and transferred to a nitrocellulose membrane (Perkin Elmer, Waltham, MA; cat. NBA085C001EA) or PVDF membrane (BioRad; cat. 1620177) for 2 h at 50 V. After blocking for 1 h in 5% nonfat dried milk (AppliChem GmbH, Darmstadt, DE; cat. A0830,0500) diluted in Tris-buffered saline (TBS) with 0.05% Tween 20 (TBST) (Sigma; cat.P7949), membranes were incubated overnight with primary antibodies at 4 °C at the dilutions indicated in Supplementary Table S1. The next day, membranes were washed 3x with TBST and incubated with the appropriate HRP-conjugated secondary antibodies for 1 h at RT. Detection of the horseradish peroxidase signal was performed using WesternBright ECL (Advansta Inc., San Jose, CA; cat. K-12045-D50) and visualised on a ChemiDoc imaging system (BioRad) using the Image Lab 6.1 software (BioRad) according to the manufacturer’s instructions. Signal intensity was quantified by ImageJ 1.53k software and normalised to the loading control indicated in the text.

### 2.10. Proliferation Assays

Cells were seeded at a density of 4 × 10^4^ cells per well in a 24-well plate and incubated overnight at 37 °C. After 24 h from seeding, cells were trypsinised and counted by the trypan blue exclusion method (time 0). Cells were then treated with the different inhibitors at the concentrations indicated in the text or vehicle controls. Every 24 h, cells were trypsinised and counted following the same method.

### 2.11. Statistical Analysis

GraphPad Prism 9 was used for the statistical analysis and for the generation of graphs. Unless otherwise specified, three biological replicates were performed. Error bars are shown as the standard error of the mean (SEM). Two-tailed paired Student’s *t*-test was used to analyse the significant difference between the two groups. One-way ANOVA analysis was selected to analyse at least three different groups when the samples showed normal distribution and equal variance. The Chi-square test was used to determine whether the difference between the observed number versus the expected number of events was significant.

## 3. Results

### 3.1. Overrepresentation of PTCH1 C-Terminal Domain Mutations in Colorectal, Endometrial and Stomach Cancers

To investigate the possibility that the CTD of PTCH1 plays a role in its tumour suppressor activity, we searched for the existence of CTD mutations in the Cancer Genome Atlas (TCGA) database. Mutations in PTCH1 were most prevalent in endometrial cancer (TCGA-UCEC), colon adenocarcinoma (TCGA-COAD) and stomach adenocarcinoma (TCGA-STAD) (Table 1). It is important to mention that the most common cancer type with loss of function mutations of PTCH1 is basal cell carcinoma of the skin (80–90%), which is not registered in the Cancer Genome Atlas. We classified the PTCH1 mutations into those likely to give a complete loss-of-function phenotype and those within the CTD (residues 1176 to 1447), which could disrupt the regulation of autophagy. PTCH1 CTD mutations are present in ~2.8% of colorectal cancers, ~2.3% gastric cancer and ~5.8% of endometrial cancers (Table 1). Interestingly, the observed number of mutations in the CTD is 2.19-fold higher than expected if each codon had an equal probability of being mutated, suggesting that the CTD is a hotspot for PTCH1 mutations in those epithelial carcinomas. 

Within all CTD mutations, three frameshift-causing indels that result in premature truncation of wild-type PTCH1 sequence accounted for around half of all mutations (Supplementary Table S2): PTCH1 S1203fs, PTCH1 R1308fs and PTCH1 Y1316fs. All three truncation mutants lack a ubiquitylation site (K1426) and the PPxY motif essential for interaction with ITCH, an E3 ubiquitin ligase that mediates PTCH1 turnover [9] and the caspase cleavage site (D1405) necessary for apoptotic induction [10,13], suggesting increased protein stability and reduced cell death.

### 3.2. Cancer-Associated PTCH1 CTD Truncations Lose Interaction with ATG101

**Figure 1:**
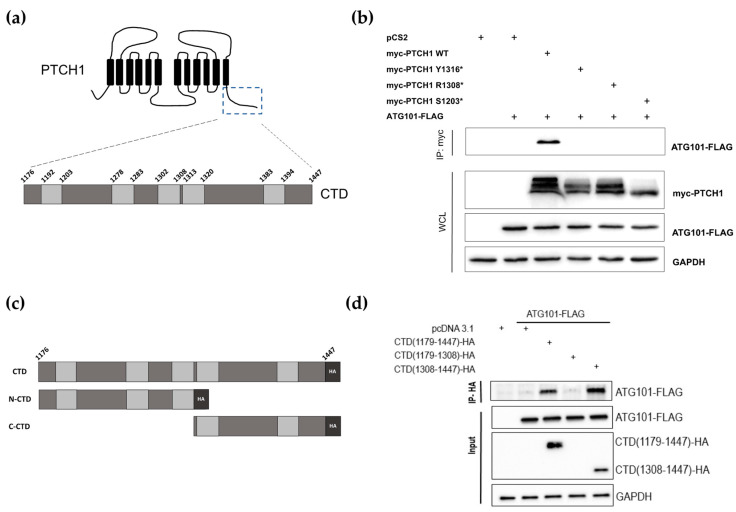
Identification of the ATG101 binding region in PTCH1. (**a**) Schematic representation of PTCH1 topological domains. The specific residues delimitating each proline-rich domain (light grey rectangles) in the C-terminal domain (CTD) are indicated in the zoomed region. (**b**) Co-immunoprecipitation of ATG101-FLAG with myc-tagged wild-type or indicated truncated mutants of PTCH1 from HEK293 cells. (**c**) Schematic representation of the isolated PTCH1 CTD construct (HA-tagged) and the two halves used in (**d**). (**d**) Co-immunoprecipitation of ATG101-FLAG with full-length CTD (1179–1447) and the C-terminal half of the CTD (1308–1447) when overexpressed in HEK293 cells. All experiments are representative of a minimum *n* = 3. All the whole western blot figures can be found in the supplementary materials.

The cytosolic CTD of PTCH1 comprises 273 amino acids and contains 5 proline-rich domains (PRDs) (Figure 1a), which are found in proteins that interact with partners containing SRC homology 3 (SH3) domains or WW domains [14]. All three most prevalent PTCH1 CTD frameshift mutants in cancer lack PRDs 4 and 5. To study whether the loss of those regions perturbs interaction with ATG101, we co-transfected HEK293 cells with myc-tagged wild-type PTCH1(1-1447), PTCH1(1-1203), PTCH1(1-1308) and PTCH1(1-1316) or empty vector, along with ATG101-FLAG. Immunoprecipitation of the PTCH1 variants using the common myc-tag showed that the interaction of ATG101 is abolished in all cancer-associated PTCH1 CTD truncation mutants (Figure 1b). To confirm that the region containing PRDs 4–5 mediates interaction with ATG101, we cloned the full CTD (1176–1447), the 1176–1308 region containing PRDs 1–3 (N-CTD) and the 1308–1447 region containing PRDs 4–5 (C-CTD) in a mammalian expression vector fused to a C-terminal HA tag (Figure 1c). Plasmids encoding the three CTD fragments were co-transfected with ATG101-FLAG in HEK293 cells, followed by immunoprecipitation of the CTD fragments and immunoblotting. Interaction of ATG101 with the full-length CTD was readily detected (Figure 1d). Interaction between ATG101 and the C-terminal half of the CTD was comparable to the full-length CTD (Figure 1d). This indicates that the 1308–1447 region of the CTD is sufficient for interaction with ATG101. Expression of the N-CTD fragment was undetectable; however, given that neither PTCH1(1-1308) nor PTCH1(1-1316) interact with ATG101, it is safe to conclude that the ATG101-interaction domain of PTCH1 is encoded in the most distal part of the CTD, within residues 1316 and 1447.

### 3.3. Cancer-Associated PTCH1 CTD Truncation Mutants Lack Autophagy Inhibitory Activity

**Figure 2:**
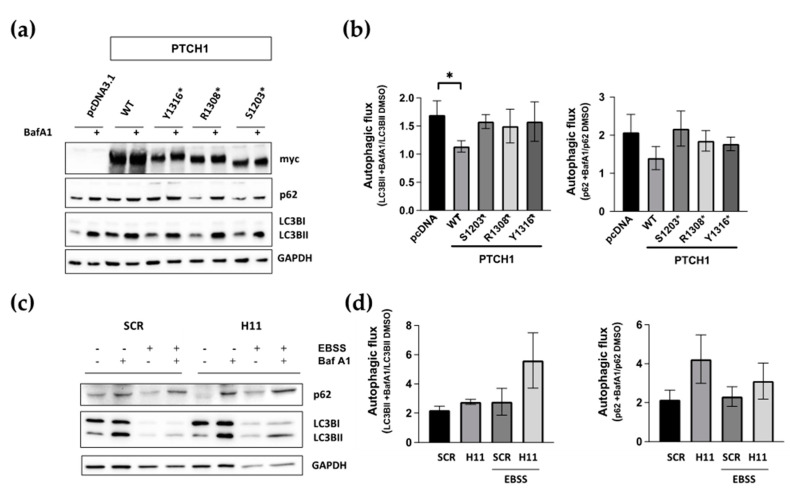
The ATG101 interacting region of PTCH1 is necessary for autophagic flux inhibition. (**a**) HEK293 cells were transfected with WT PTCH1 or the indicated variants. After 20 h, 100 nM Bafilomycin A1 (Baf A1) or the same amount of DMSO were added for an additional 4 h, before lysing the cells and determining the levels of p62/SQSMT1 (p62) and LC3BI and II; (**b**) Autophagic flux determination from densitometric analysis of the change in the level of LC3BII and p62 normalised to GAPDH in cells expressing the different PTCH1 mutants in the presence and absence of bafilomycin A1. Data represent the mean +/− SEM of 3 biological replicates, * *p* < 0.05; (**c**) Autophagy markers levels in CRISPR/Cas9 engineered HEK293 cells expressing endogenous wild-type PTCH1 (SCR) and PTCH1 D1222fs (H11) under standard growth conditions or after 4 h of nutrient starvation (EBSS); (**d**) Autophagic flux determination from densitometric analysis of the change in the level of LC3BII and p62 normalised to GAPDH in SCR and H11 cells in the presence and absence of bafilomycin A1. Data represent the mean +/− SEM of 3 biological replicates. All the whole western blot figures can be found in the supplementary materials.

Next, we investigated whether the inability of the PTCH1 CTD mutants to associate with ATG101 could lead to impairment of PTCH1′s role to repress autophagic flux. During completion of autophagy, autophagosome-localised LC3B-II and the ubiquitylated cargo adaptor p62/SQSMT1 are degraded along with the cargo. Inhibition of lysosomal hydrolases or autophagosome-lysosome fusion results in a large accumulation of LC3B-II and p62 when autophagic flux is high and in a smaller accumulation of both markers when the autophagic flux is slow or blocked [15]. We have previously used the inhibitors bafilomycin A1 (BafA1) and chloroquine to demonstrate that overexpressed or endogenous PTCH1 functions inhibit autophagic flux [5]. To determine whether the CTD truncation mutants are defective in autophagy inhibitory activity, we followed the changes in LC3B-II and p62 in HEK293 cells expressing myc-tagged wild-type PTCH1(1-1447), PTCH1(1-1203), PTCH1(1-1308), or empty vector in the absence or presence of BafA1 during the last 4 h. As shown in Figure 2a,b, none of the three PTCH1 truncations reduced autophagic flux, unlike wild-type PTCH1(1-1447). This result was expected since they cannot interact with ATG101.

To confirm that truncations of the CTD of PTCH1, when expressed from the endogenous locus, also lose the autophagy inhibitory capacity, we introduced indel mutations in endogenous PTCH1 after S1223 using CRISPR/Cas9 in HEK293 cells. The frameshift takes place after D1222, between the most common cancer indels at S1203 and R1308, due to gRNA design (Supplementary Figure S1). The autophagic flux of PTCH1 CTD mutant cells (H11) was slightly, but clearly, increased compared to isogenic control cells expressing WT PTCH1 (SCR) (Figure 2c,d). The enhanced effect of Bafilomycin A1 was observed on basal autophagy when cells were grown in a complete medium, as well as when induced by amino acid starvation by incubation in EBSS. Taken together, these results indicate that the most common cancer-associated CTD truncation mutants are loss-of-function for autophagy regulation by loss of interaction with ATG101.

### 3.4. Truncation of the CTD in Endogenous PTCH1 in Colorectal Cancer Cells Increases Autophagic Flux and Dysregulates Expression of Autophagy-Related Proteins

**Figure 3:**
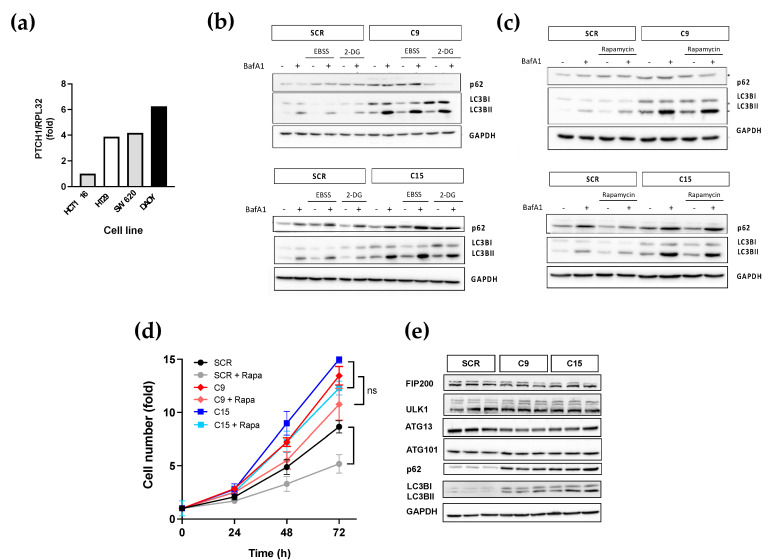
Colon cancer cells with a PTCH1 truncation exhibit dysregulated autophagic flux. (**a**) Normalised PTCH1 transcript levels in 3 colon cancer cell lines in comparison to DAOY cells determined by qPCR; (**b**) Autophagic flux markers changes in response to nutrient depletion (EBSS) or glycolysis inhibition (25 mM of 2-DG) in SW620 clones C9 and C15 expressing endogenous PTCH1 1223fs (C9) compared to isogenic cells expressing WT PTCH1 (SCR); (**c**) Autophagic flux markers changes in response to treatment with 0.5 μM rapamycin (RAP) in SW620 clones C9 and C15 expressing endogenous PTCH1 1223fs (C9) or WT PTCH1 (SCR); (**d**) Fold change in cell number of SCR, C9 and C15 cells over time in the absence or presence of 0.5 μM rapamycin (Rapa). Data shows mean +/− SEM of 3 biological replicates, * *p* < 0.05, ns: not significant; (**e**) Protein expression levels of ULK complex subunits, p62 and LC3B in untreated SW620 cells expressing WT PTCH1 (SCR) or PTCH1 S1223fs (C9 and C15). Lysates from 3 different passages were compared for each clone. All the whole western blot figures can be found in the supplementary materials.

Since the previous findings were obtained with immortalised normal cells, we next investigated whether frameshift-causing mutations of PTCH1 could increase the autophagic flux in colon cancer cells. Thus, indel mutations after S1223 were engineered in SW620 cells by CRISPR/Cas9 technology to generate isogenic cancer cell line pairs differing only in their PTCH1 mutational status. SW620 cell lines were chosen solely based on the expression level of endogenous PTCH1, as determined by qPCR (Figure 3a). We performed experiments on two single-cell derived clones harbouring different frameshift-causing indels (C9 and C15) alongside the isogenic cells expressing wild-type PTCH1 (SRC) to lower the risk of picking up a non-specific phenotype. The SW620 isogenic cell lines are fully characterised in other articles [16,17].

Analysis of basal autophagic flux in isogenic cell lines showed that truncation of the CTD of endogenously expressed PTCH1 in two independent single cell-derived clones results in an apparent increase in autophagic flux, as determined by a larger magnitude accumulation of LC3B-II in response to Bafilomycin A1 (Figure 3b). The increased autophagic flux was observed in basal conditions and after autophagy induction by rapamycin or amino acid starvation (both resulting in mTORC1 inhibition) and by 2-deoxyglucose (which leads to AMPK activation) (Figure 3b,c). Furthermore, truncation of PTCH1 in C9 and C15 cells increased their proliferation rate, which was not significantly reduced by autophagy induction with rapamycin. In contrast, the isogenic cells with WT PCTH1 (SRC) had a ~40% decrease in proliferation rate in the presence of rapamycin (Figure 3d). However, the increase in autophagic flux was accompanied by increased protein expression levels of p62/SQSTM1 and LC3B in the PTCH1 mutant clones (Figure 3e). These findings suggest that mutations of the CTD of PTCH1 found in cancer cells dysregulate macroautophagy.

### 3.5. PTCH1 CTD Truncation Enhances Proliferation under Nutrient Starvation

**Figure 4:**
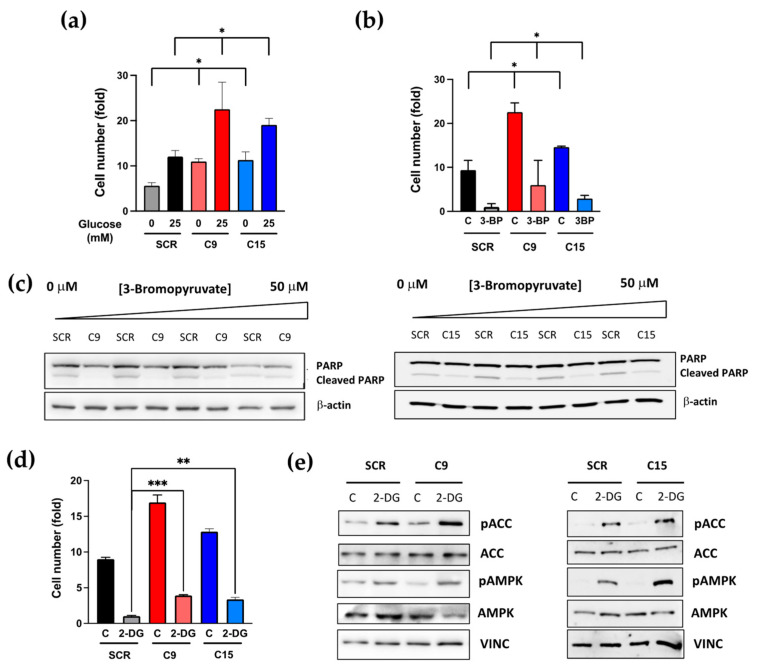
Colon cancer cells containing a PTCH1 CTD truncation exhibit increased proliferation under nutrient stress conditions compared to cells expressing WT PTCH1 (SCR) (**a**) Fold change in cell number of SCR, C9 and C15 cells after 72 h cultured in the presence of glucose (25 mM) or in the absence of glucose (0 mM). Initial seeding = 20,000 cells/cm^2^. Data shows mean +/− SEM of 3 biological replicates, * *p* < 0.05; (**b**) Fold change in cell number of SCR, C9 and C15 cells after 72 h cultured in the presence of 50 μM 3-bromopyruvic acid (3-BP) or vehicle (C). Initial seeding = 20,000 cells/cm^2^. Data shows mean +/− SEM of 3 biological replicates,* *p* < 0.05; (**c**) Total and cleaved PARP levels in SCR, C9 and C15 cells after 48 h treatment with increasing doses of 3-BP; (**d**) Fold change in cell number of SCR, C9 and C15 cells after 72 h cultured in the presence of 25 mM 2-deoxygluse (2-DG) or vehicle (C). Initial seeding = 20,000 cells/cm^2^. Data shows mean +/− SEM of 3 biological replicates, ** *p* < 0.01, *** *p* < 0.001; (**e**) Phosphorylation of AMPK and ACC in SCR, C9 and C15 cells after 2 h treatment with 25 mM 2-DG. All the whole western blot figures can be found in the supplementary materials.

Given that autophagy is a short-term adaptive response to nutrient starvation, by providing biosynthetic building blocks through the recycling of amino acids and lipids, we investigated whether the proliferation of cancer cells carrying the PTCH1 CTD mutation were more robust than the isogenic control cells under energy stress. We first compared their ability to proliferate under suboptimal glucose supplementation. The proliferation of both wild-type and PTCH1 mutant cells was reduced at reduced glucose concentration. However, cells mutant for PTCH1 displayed a proliferative advantage even in the absence of added glucose, with their cell number comparable to SCR cells growing at high glucose levels (25 mM) (Figure 4a). The PTCH1 mutant cells also showed a proliferative advantage when grown in the presence of glycolysis inhibitors. The addition of 3-bromopyruvic acid (3-BP) dose-dependently reduced proliferation in WT and mutant cells (Supplementary Figure S2), but the latter still proliferated in the presence of 50 μM 3-BP (Figure 4b) and showed a diminished poly-ADP ribosyl polymerase (PARP) cleavage response to 3-BP (Figure 4c), indicative of reduced apoptosis. PTCH1 mutant cells were also more resistant to 2-deoxyglucose (2-DG), another glycolysis inhibitor (Figure 4d), and this was accompanied by enhanced activation of AMPK and its downstream substrate ACC (Figure 4e).

Altogether, these data suggest that mutation of the PTCH1 CTD enhances the nutrient stress adaptive response of autophagy and reduces dependency on glycolysis.

## 4. Discussion

The Hh family receptor PTCH1 is a well-characterised tumour suppressor. A large number of loss-of-function mutations distributed along most exons and intron-exon boundaries result in no expression or expression of inactive PTCH1 that cannot repress Smoothened in the absence of Hh ligands. This type of mutation results in constitutive activation of canonical Hh signalling and is the leading cause of basal cell carcinoma of the skin [18] and a large fraction of Shh-type medulloblastoma [19]. A different type of PTCH1 mutation in its cytosolic C-terminal domain (CTD) has been reported in squamous cell carcinomas of the skin and upper GI tract. However, most of the CTD of PTCH1 is dispensable for Smoothened repression by PTCH1 [8]. In this study, we present evidence for the first time that mutations in the CTD of PTCH1 prevent interaction with ATG101 and enhance the proliferation and survival of cancer cells under nutrient stress.

We have previously reported that the CTD of PTCH1 inhibits autophagy through interaction with the ULK complex subunit ATG101 [5]. We hypothesised that this novel function could contribute to the tumour suppressor functions of PTCH1 and searched for evidence of somatic mutations in cancer that only affect this domain. Here, we report a 2.3–5.85 % prevalence of mutations in the PTCH1 CTD in the stomach, colorectal and endometrial cancers, with three frequent frameshift spots that result in truncation of the wild-type sequence after S1203, R1308 and Y1316. With the aim to determine whether the truncation mutants retain interaction with ATG101, we performed co-immunoprecipitation studies and demonstrated that none of the cancer-associated mutants binds ATG101. In a separate set of experiments using fragments of the PTCH1 CTD, we showed that the most distal half of the CTD, containing two proline-rich domains, is necessary and sufficient for interaction with ATG101.

The loss of physical interaction of the PTCH1 mutants with ATG101 was associated with a reduction in the ability of the mutants to inhibit autophagic flux in conditions of overexpression. However, truncation of endogenous PTCH1 in a residue between the cancer-associated truncations (S1223) using CRISPR/Cas9 also resulted in enhanced autophagic flux both in nutrient plenty, as well as under nutrient starvation conditions. This suggests that the endogenous expression level of PTCH1 is sufficient to modulate autophagic flux.

To further validate this conclusion, we engineered the CTD truncation at S1223 in a colorectal cancer cell line without mutations in PTCH1 by CRISPR/Cas9. Two different clones with indel mutations in the CTD of PTCH1 were used to compare to isogenic control cells in several assays. As predicted, the mutation in the PTCH1 CTD was associated with increased basal and induced autophagic flux; however, we observed a more profound alteration in the regulation of autophagy by altered expression of some autophagy-related proteins. Future studies will investigate the mechanisms underlying this profound dysregulation, which cannot be explained only by the loss of the PTCH1:ATG101 interaction.

Our findings also indicate that cancer cells with truncation of the PTCH1 CTD display increased fitness than cells carrying wild-type PTCH1. This is demonstrated by an increased rate of proliferation in nutrient plenty conditions and by a much-reduced sensitivity to glycolysis inhibitors. It is possible that the enhanced autophagic flux in those cells improves their nutrient stress adaptability and increases metabolic flexibility, although the role of additional alterations as a consequence of the PTCH1 CTD mutation cannot be ruled out.

## 5. Conclusions

Our findings suggest that mutations in the CTD of PTCH1, relatively common in some epithelial cancer types, contribute to cancer cell fitness by loss of PTCH1 regulation of autophagy and increase in metabolic flexibility under nutritional stress. While our observations do not prove that the CTD of PTCH1 encodes a tumour suppressor function, as we did not demonstrate sufficiency to cause transformation, they indicate an important regulatory role of key cancer cell properties.

## Figures and Tables

**Table 1:** Frequency of total and CTD-specific PTCH1 mutations in three cancer sites according to the TCGA database.

Cohort (Site)	*PTCH1* Mutations (%)	*PTCH1* CTD Mutations (%)	Enrichment of CTD Mutations (fold) ^1^
TCGA-UCEC	62/512 (12.11%)	30/512 (5.85%)	2.56
TCGA-COAD TCGA-STAD All combined	31/427 (7.26%) 33/434 (7.60%) 126/1373 (9.18%)	12/427 (2.8%) 10/434 (2.3%) 52/1373 (4.08%)	2.07 1.6 2.19 *

^1^ prevalence of CTD mutations normalized to the expected frequency based on full length PTCH1 and CTD cDNA length. * *p* < 0.0001 two-tailed; Chi-squared test (χ^2^ = 41.622, 1 df).

## Data Availability

All data related to this study is presented in this manuscript; however, any reasonable request can be directed to N.A.R.-D.G.

## Supplementary Materials

The following is available online at https://www.mdpi.com/article/10.3390/cancers15020369/s1, Figure S1: CRISPR/Cas9 design to introduce indel mutations in endogenous PTCH1; Figure S2: Colon cancer cells containing a PTCH1 CTD truncation exhibit increased proliferation after increasing doses of 3-bromopyruvic acid (3BP); Table S1: Antibodies used in this study; Table S2: PTCH1 CTD mutations present in the TCGA-CDG projects COAD, STAD and UCEC.
