# Supplementary material for: Partial Truncation of the C-Terminal Domain of PTCH1 in Cancer Enhances Autophagy and Metabolic Adaptability
Source: Cancers (Basel). 2023 Jan 6;15(2):369. doi: 10.3390/cancers15020369 (PMC9856372; doi:10.3390/cancers15020369)
Supplement: Supplementary file 1 [file cancers-15-00369-s001.zip › cancers-2107013-supplementary.pdf]

Supplementary Table S1. Antibodies used in this study.

**Antibody**[1]	**Vendor**	**Catalogue**	**dilution**
anti-myc (M)	Cell Signalling Technology	2276	1:1000
anti-myc (R)	Proteintech	16286-1-AP	1:1000
anti-FLAG (M)	Proteintech	66008-4-IG	1:1000
anti-FLAG (R)	Cell Signalling Technology	2368	1:1000
anti-HA (M)	Proteintech	66006-1-IG	1:1000
anti-HA (R)	Proteintech	51064-2-AP	1:1000
anti-LC3B (R)	Cell Signalling Technology	3868	1:1000
anti-p62/SQSMT1 (R)	Cell Signalling Technology	5114	1:1000
anti-PARP 9542 (R)	Cell Signalling Technology	9542	1:1000
anti-Atg101 (R)	Cell Signalling Technology	13492	1:1000
anti-Atg13 (R)	Cell Signalling Technology	6940	1:1000
anti-ULK1/2 (R)	Cell Signalling Technology	4773	1:1000
anti-FIP200 (R)	Cell Signalling Technology	12476	1:1000
anti-alpha-Tubulin (M)	Proteintech	66031-1-IG	1:1000
anti-β-actin (M)	Sigma-Aldrich	A3853	1:5000
anti-ACC (R)	Cell Signalling Technology	3676	1:1000
anti-phospho-ACC(S79) (R)	Cell Signalling Technology	3661	1:1000
anti-AMPKα (R)	Cell Signalling Technology	5832	1:1000
anti-phospho-AMPKα(T172) (R)	Cell Signalling Technology	2535	1:1000
anti-vinculin (M)	Santa Cruz Biotechnology	sc73614	1:3000
anti-GAPDH-HRP (M)	Proteintech	HRP-60004	1:3000
anti-rabbit IgG HRP (G)	BioRad	172-1019	1:3000
anti-mouse IgG (HL)-HRP (G)	Bethyl	A90-116P	1:3000

[1] Host: M, mouse; G, goat; R, rabbit

Supplementary Table S2. PTCH1 CTD mutations present in the TCGA-CDG projects COAD, STAD and UCEC.

DNA change	Type	Consequences	Number
**Chr9:g.95449267delG**	**Deletion**	**Frameshift (S1203Afs*52)**	**9**
**Chr9:g.95449266 95449267insG**	**Insertion**	**Frameshift (S1203Qfs*17)**	**2**
**Chr9:g.95447335delG**	**Deletion**	**Frameshift (R1308Efs*64)**	**7**
**Chr9:g.95447334 9544733insG**	**Insertion**	**Frameshift (R1308Qfs*17)**	**2**
**Chr9:g.95447311delG**	**Deletion**	**Frameshift (Y1316Tfs*56)**	**5**
Chr9:g.95446982G>A	Substitution	Missense (S1425L)	2
Chr9:g.95449298C>T	Substitution	Missense (R1192H)	2
Chr9:g.95446981C>T	Substitution	Synonymous (S1425=)	1
Chr9:g.95447092C>G	Substitution	Synonymous (G1388=)	1
Chr9:g.95449081G>A	Substitution	Synonymous (F1264=)	1
Chr9:g.95449158G>A	Substitution	Missense (R1239W)	1
Chr9:g.95447439C>T	Substitution	Missense (E1273K)	1
Chr9:g.95446945G>A	Substitution	Synonymous (C1437=)	1
Chr9:g.95449204C>T	Substitution	Synonymous (S1223=)	1
Chr9:g.95447410C>T	Substitution	Synonymous (P1282=)	1
Chr9:g.95447401C>A	Substitution	Missense (Q1285H)	1
Chr9:g.95447450A>G	Substitution	Missense (V1269A)	1
Chr9:g.95449205G>A	Substitution	Missense (S1223L)	1
Chr9:g.95449108G>A	Substitution	Synonymous (I1255=)	1
Chr9:g.95447183C>T	Substitution	Missense (G1358D)	1
Chr9:g.95449132C>T	Substitution	Synonymous (A1247=)	1
Chr9:g.95447232G>A	Substitution	Missense R1342C	1
Chr9:g.95447069delC	Deletion	Frameshift (G1396Dfs*56)	1
Chr9:g.95447140C>A	Substitution	Synonymous (T1372=)	1
Chr9:g.95447103G>A	Substitution	Missense (P1385S)	1
Chr9:g.95447141G>A	Substitution	Missense (T1372M)	1
Chr9:g.95449309G>A	Substitution	Synonymous (N1188=)	1
Chr9:g.95447112G>A	Substitution	Missense (H1382Y)	1
Chr9:g.95449851G>T	Substitution	Missense (P1180Q)	1
Chr9:g.95447319G>A	Substitution	Missense (P1313S)	1

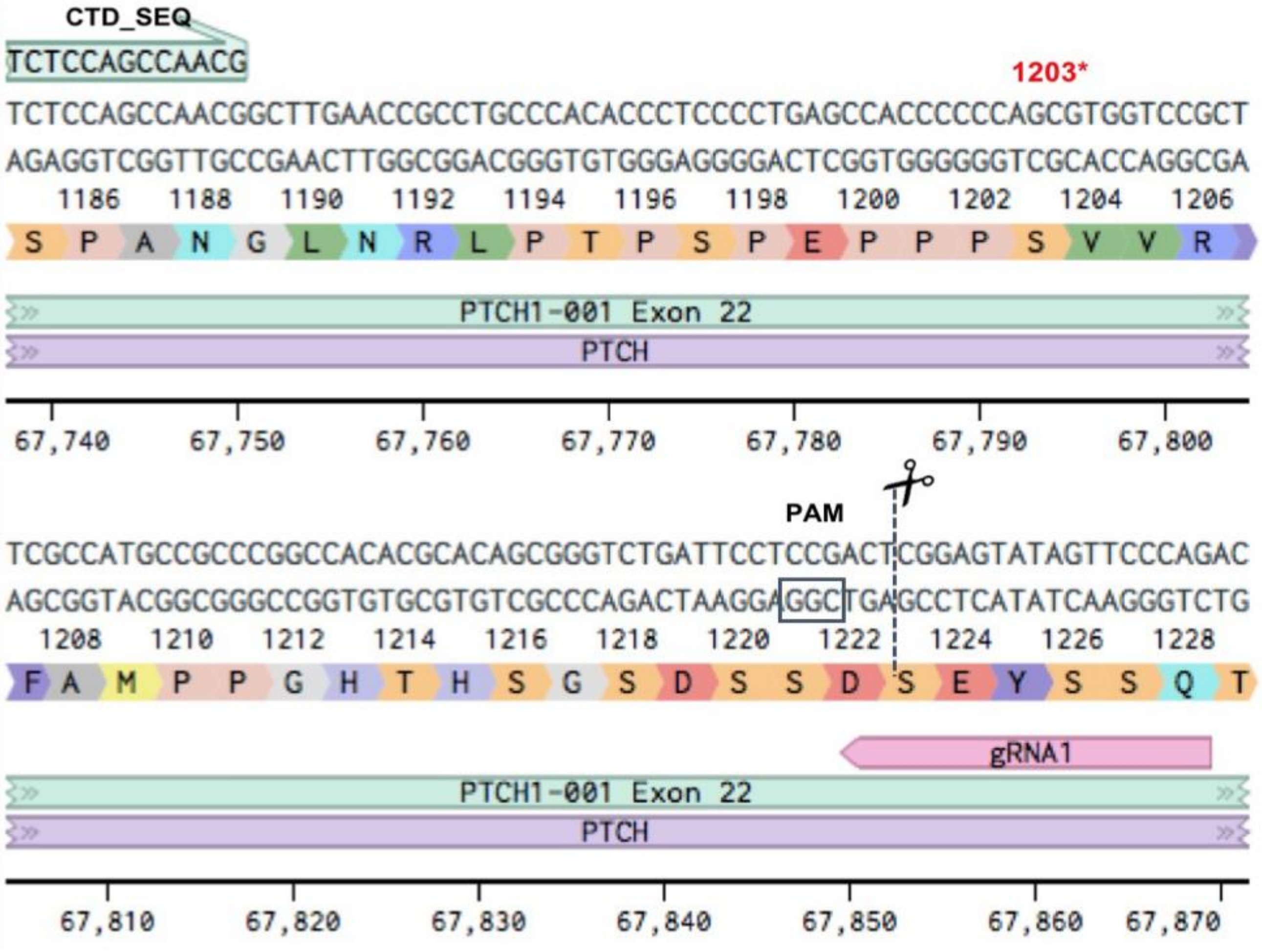

Figure S1. CRISPR/Cas9 design to introduce indel mutations in endogenous PTCH1. 1203* (in red) represents one of the most common cancer indels.

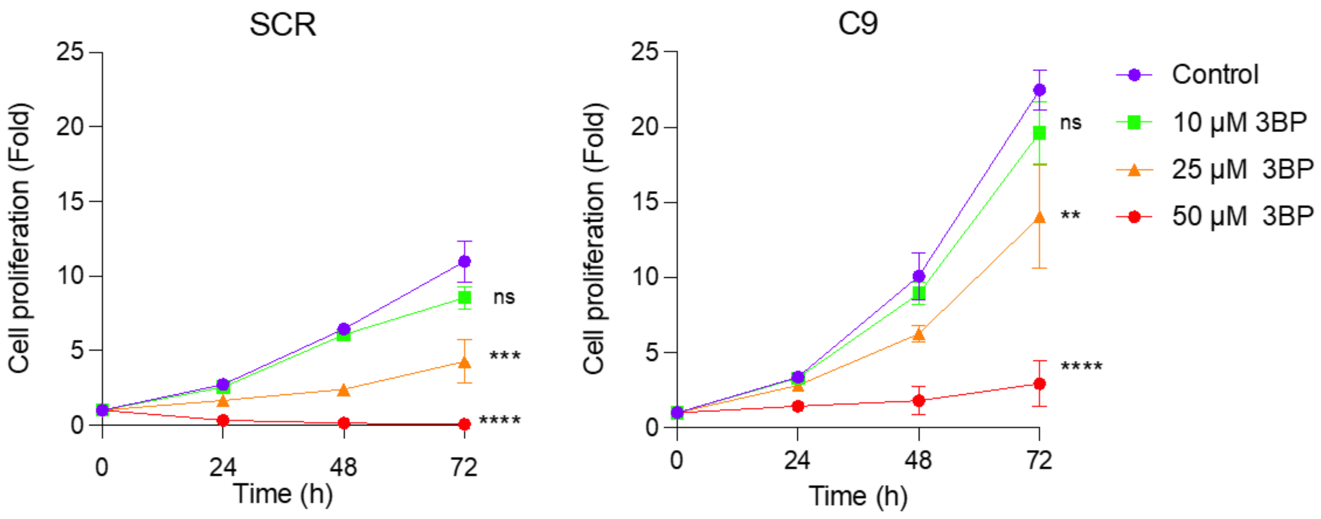

Figure S2. Colon cancer cells containing a PTCH1 CTD truncation exhibit increased proliferation after increasing doses of 3-bromopyruvic acid (3BP). Fold change in cell number of SCR and C9 cells over time in the presence of different concentrations of 3BP, as indicated in the figure. Initial seeding = 20,000 cells/cm^2. Data shows mean +/- SEM of 3 biological repeats, **<0.01, ***<0.001, ****<0.0001, ns: not significant.

Whole western blots related to main figures:

Figure 1.

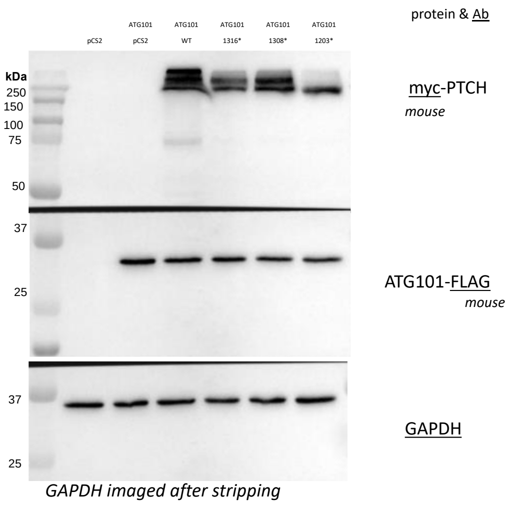

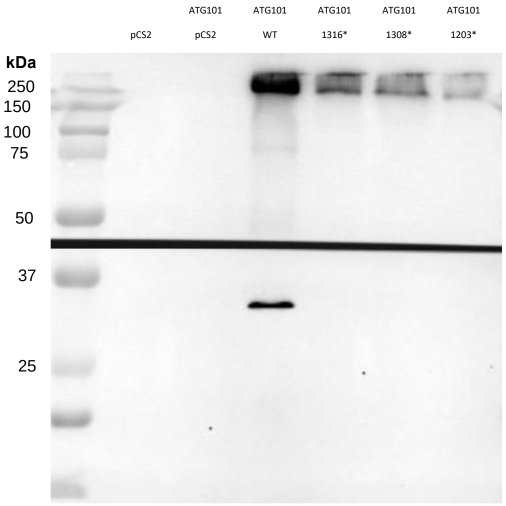

Figure 2.

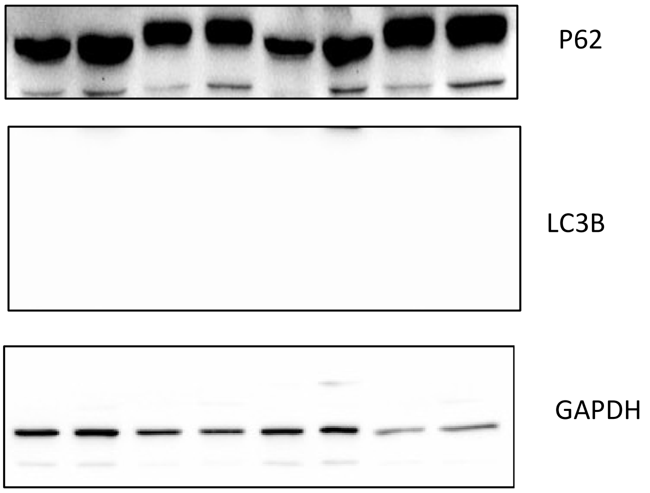

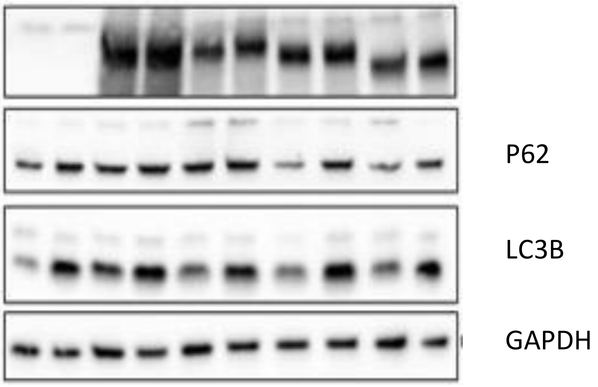

Figure 3.

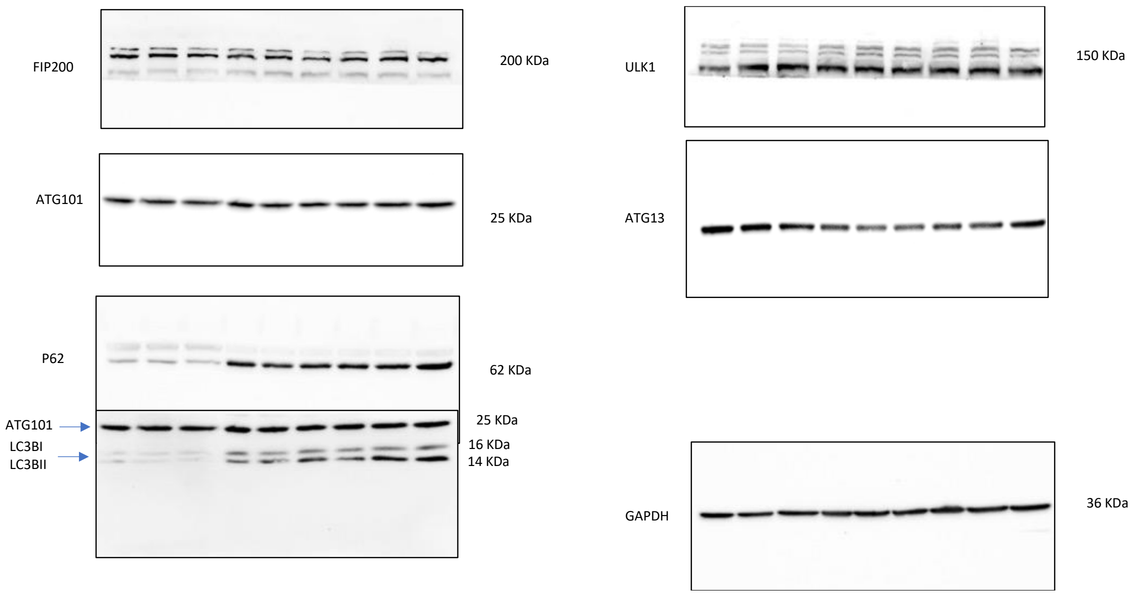
FIP200
200 KDa
ULK1
150 KDa
ATG101
25 KDa
ATG13
P62
62 KDa
ATG101
25 KDa
LC3BI
LC3BII
16 KDa
14 KDa
GAPDH
36 KDa

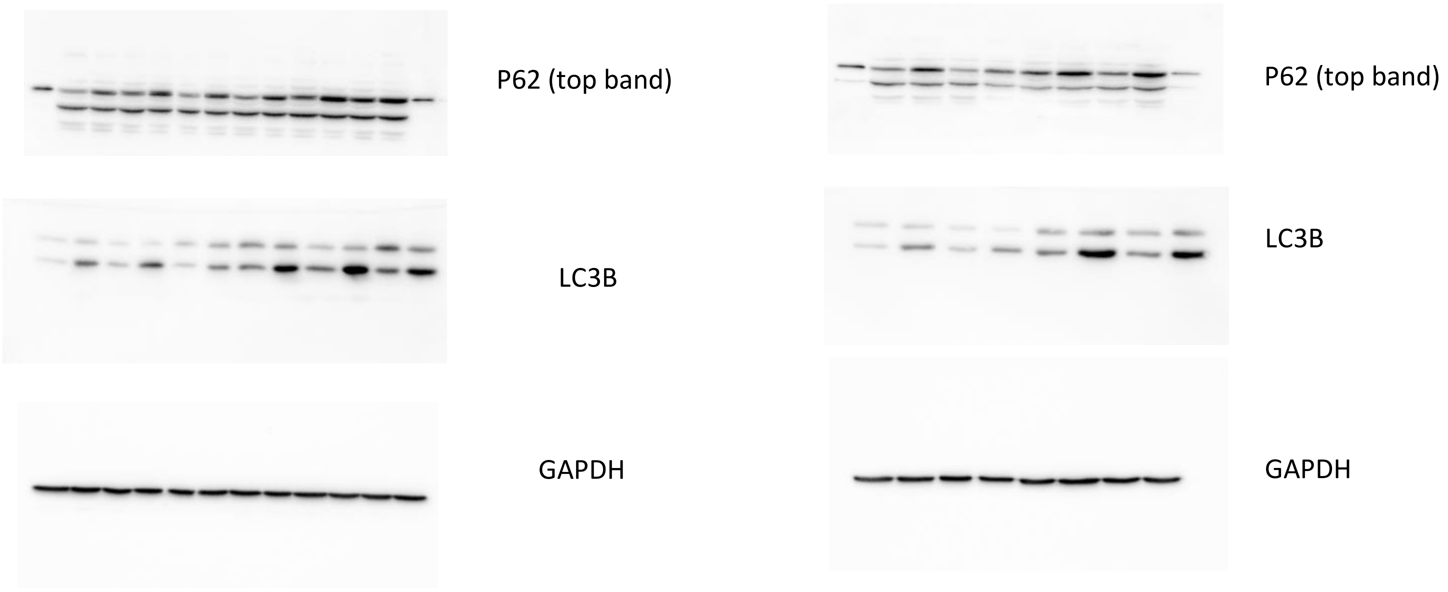
P62 (top band)
P62 (top band)
LC3B
LC3B
GAPDH
GAPDH

Figure 4.

pACC
ACC
pAMPK
AMPK
VINC

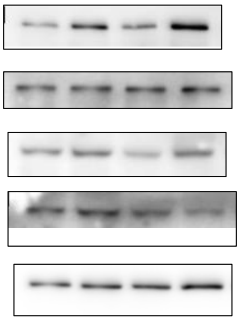

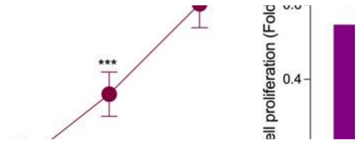

PARP/
Cleaved PARP
actin

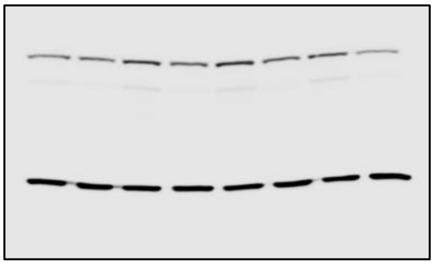

PARP
Cleaved PARP
β-actin

Top gel cut and exposed for longer

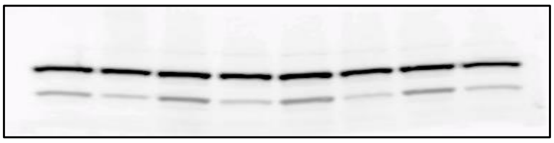

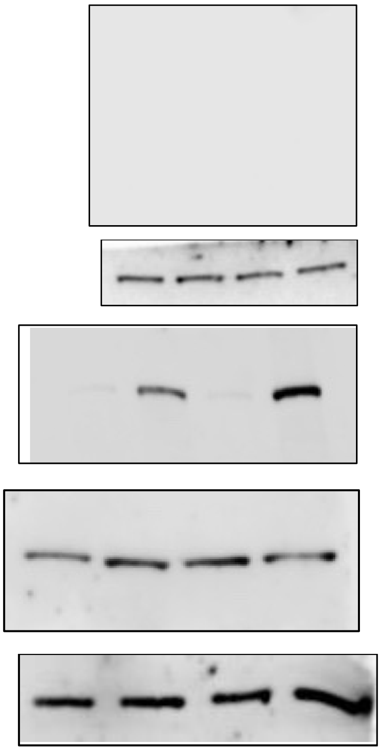

pACC
ACC
pAMPK
AMPK
vinculin
